# Total body water by BIA in children and young adults with normal and excessive weight

**DOI:** 10.1371/journal.pone.0239212

**Published:** 2020-10-08

**Authors:** Tej K. Mattoo, Hong Lu, Eric Ayers, Ronald Thomas

**Affiliations:** 1 Department of Pediatrics, Wayne State University School of Medicine, Detroit, Michigan, United States of America; 2 Department of Internal Medicine, Wayne State University School of Medicine, Detroit, Michigan, United States of America; San Raffaele Roma Open University, ITALY

## Abstract

**Background:**

Estimation of total body water (TBW) is essential for clinical care.

**Objective:**

Evaluation of changes in TBW by bioelectrical impedance analysis (BIA) in children and young adults with excessive weight.

**Design:**

Data was collected in individuals aged 3–21 years with normal (n = 202) or excessive body weight (n = 133). The BIA results from individuals with normal weight were compared with two previously published studies in children by isotope dilution methods.

**Results:**

Individuals with excessive weight had a higher mean TBW (27.87 L, SE 0.368) for height and age as compared to individuals with normal weight (23.95 L, SE 0.298), P<0.001. However, individuals with excessive weight had lower mean TBW (24.93 L, SE 0.37) for weight and body surface area (BSA) as compared to individuals with normal weight (26.94 L, SE 0.287), P<0.001. Comparison with two previously published studies showed no significant differences in mean TBW with one ((p = 1.00) but a significant difference with another study (p = 0.001).

**Conclusions:**

Individuals with excessive weight had 16.5% higher mean TBW for height and age and 7.4% lower TBW for weight and BSA as compared to normal weight individuals. Our study validates the feasibility of data collection in pediatric outpatient setting by BIA.

## Introduction

Estimation of total body water (TBW) is integral to clinical care. It has significant implications for patient care that include dosing of medications, assessment and treatment of dehydration, fluid and energy requirements for parenteral nutrition, and dialysis prescriptions. Although the amount of TBW increases with growth from birth to adulthood, its fraction as a percentage of body weight decreases from about 80% at birth [1] to about 60% in adult men and 50% in adult women [1, 2].

There are several methods for body water estimation [3–7]. Of these, the isotope dilution technique is considered as sufficiently accurate and is used as a reference method for body water estimation. The major limitation with any of these methods is that they can be expensive and time consuming, need appropriate institutional resources, and are not possible in routine outpatient clinic setting, particularly in pediatric patients.

Bioimpedance analysis (BIA) is an alternative method for quantifying body water and its compartmental distribution. A large number of studies have validated the accuracy of BIA for body water estimation by comparing results with simultaneously collected data by dilution methods [5, 8–10]. Studies on TBW by dilution methods are not possible during routine outpatient clinic visits. BIA, in spite of some limitations, offers a potential substitute that deserves further exploration. Very little has been published on a quantitative comparison of TBW for age, weight, height or the body surface area (BSA) in children with normal and excessive weight and, to the best of our knowledge, no study has compared data collected by BIA with the historical data by dilution methods in children. The main objective of our study was to evaluate weight based changes in TBW noninvasively by BIA in ambulatory clinic settings in children and young adults. For validation of our data, we compared our results with two previously published studies in children by dilution methods.

## Patients and methods

A total number of 335 BIA studies were done in 312 (93.1%) pediatric patients or their siblings seen in Nephrology Clinic at the Children’s Hospital of Michigan and Med-Peds clinic of the University Physician Group over a period of 18 months. In some participants data was collected more than once and the minimum time interval between repeat studies in the same individual was six months. Children and young adults aged 3 years to 21 years with normal or increased body weight were included in the study. Excluded from the study were patients with diabetes, dehydration, hypertension with or without medications, internal defibrillator or pacemakers, missing limb, medications that affect body water content such as diuretics and glucocorticoids, menstruation, pregnancy, moderate exercise, consumption of a big meal within 2 hours before the procedure, and chronic kidney disease or any other co-morbid condition.

**Fig 1:**
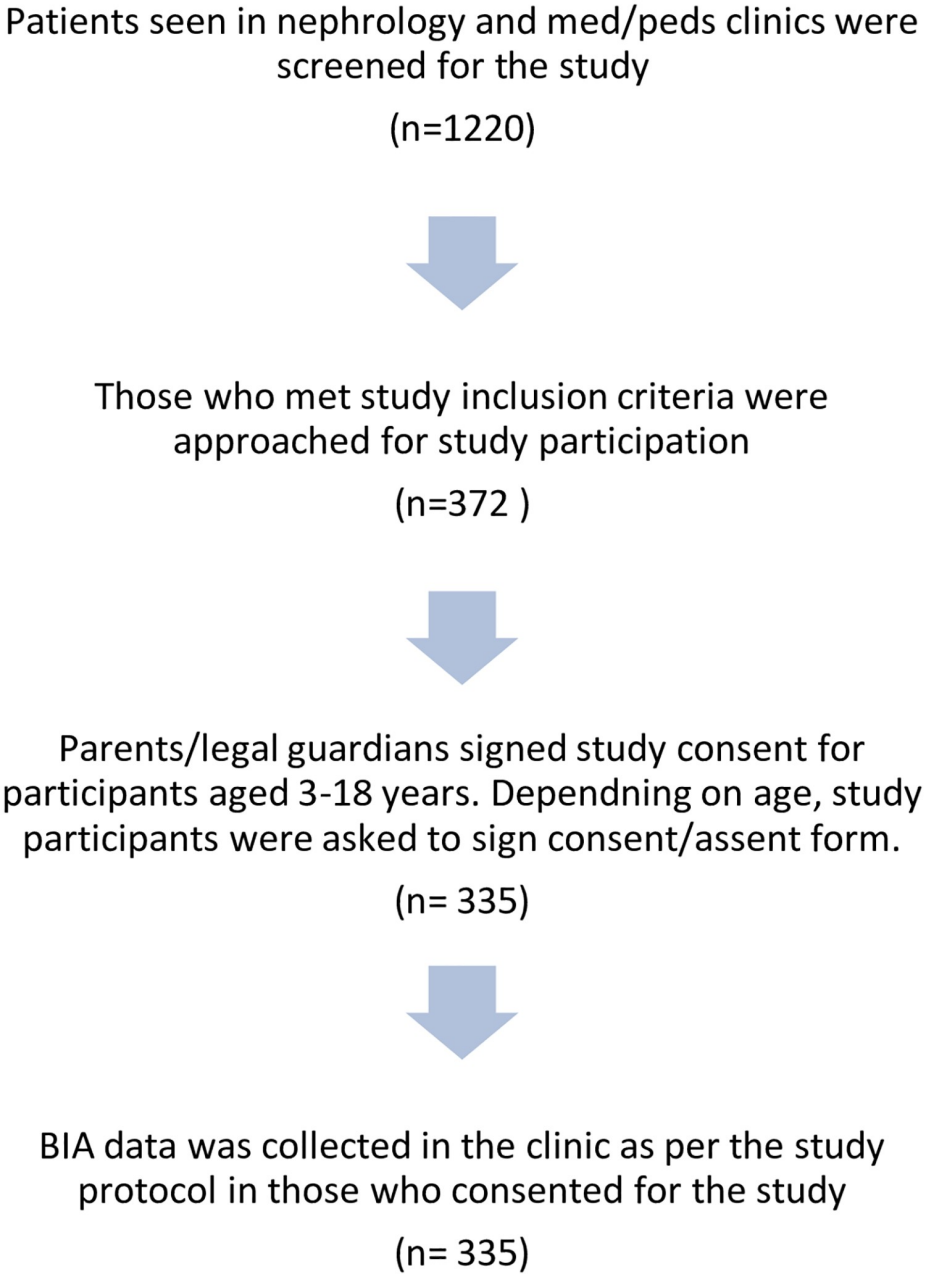
Selection of study participants for bioimpedance analysis (BIA) data collection.

The study was approved by the Wayne State University Institutional Review Board. Parents or legal guardians of study participants aged 3–18 years had to sign study consent and those aged 13–18 years had to sign an assent form as well. Participants older than 18 years signed study consent by themselves. The study participant selection process is shown in Fig 1.

Height and weight were measured according to the standardized procedure with shoes and jackets off and only with light clothing [11]. Blood pressure was measured in accordance with the AAP guidelines [12] with manual confirmation of high readings by oscillometric methods.

### BIA measurement

Direct segmental multi-frequency bioelectrical impedance analysis device InBody s10 (InBody Co. Ltd) was used for the study. Measurements were made in temperature controlled offices, on examination tables with patient sitting with legs hanging- arms and legs abducted. Before each measurement, study participants were asked to void and sit down for 10 to 15 minutes on the examination table. Touch-type electrodes were placed on participants’ feet near ankle and hands near wrist. A current frequency of 50, 100, 500 and 1000 kHz at 5 segments (right arm, left arm, right leg, left leg, and trunk) was applied for a total period of about minute and a half until completion of recording, which was indicated on the screen and with a beeping sound. Two study investigators used the same BIA machine for data collection from all study participants.

To reduce the risk of measurement error, most patients had three back to back measurements for each study and the mean of three reading was used for data analysis. Studies with coefficient of variation of more than 5% between the three readings were excluded from data analysis. Body weight was defined on the basis of body mass index (BMI) as normal weight BMI<85th percentile), overweight (BMI >85th to 95th percentile), and obesity (BMI >95th percentile) [13]. Excessive weight in our study includes individuals with overweight as well as obesity.

We compared our BIA results with two previously published studies by dilution method in children [14, 15].

### Statistical analysis

To express precision and repeatability of BIA measurements, the coefficient of variation was calculated and expressed as a percentage, defined as the ratio of the standard deviation to the mean. Studies with a coefficient of variation of more than 5% between the three readings were excluded from data analysis. Descriptive statistics were reported for both normal and overweight children.

We compared our BIA results with two previously published studies by dilution method in children [14, 15]. The mean of two or three BIA readings for each participant was used for data analysis and the coefficient of variation was calculated and reported for each. Demographic data from study participants was reported using frequencies procedures. Scatterplot graphs and best fit regression equations were reported separately for normal and overweight children, as well as gender. Bioimpedance data obtained from normal weight children was compared to two studies using the isotope dilution methodology. Regression equations were calculated for each study and standardized residual values computed. Median differences in standardized residuals between study groups were examined using a non-parametric Kruskal-Wallis procedure, with pair wise comparisons conducted with a non-parametric Mann Whitney U procedure. Accuracy of prediction models between studies were assessed using explained variance (R2), mean square error (MSE), square root of MSE, and average absolute percent error. The study was approved by our Institutional Review Board. All statistical procedures were conducted using NCSS statistical software Version 11.0.

### Study results

A total of 335 studies were done in 312 (93.1%) study participants, one time only in 291 (86.9%), twice in 19 and three times in two participants. Their ages at the time of study ranged from 3 to 21 years with a mean age of 11.0 ± 4.4 years and the mean weight of 47.5 ± 25.9 kg. The gender ratio was almost equal with 173 (52%) males and 162 (48%) females. Of the total number of 335 studies, 202 (60%) were in normal weight and 133 (40%) were in individuals with excessive weight. There were no significant differences in age, gender, race, and height between normal weight and overweight/obese groups. As expected, weight, BMI and BSA are significantly different between the two groups. Demographic details are reported in Table 1.

**Table 1:** Demographic data on study participants.

	Total (n = 335)	Normal weight (n = 202)	Overweight & Obese (N = 133)	P value
Age (years) (mean± SD)	11.0 ± 4.39	10.95 ± 4.5	11.2 ± 4.2	0.92
Range	(3–21)	(3.1–21)	(4–19.5)	
Gender				
• Male	173 (51.6%))	95 (47%)	78 (58.6%)	0.048
• Female	162 (48.4%)	107 (53%)	55 (41.4%)	
Race				
• White	90 (26.8%)	54 (26.7%)	36 (27%)	0.78
• African American	98 (29.3%)	54 (26.7%)	44 (33.1%)	
• Other*	147 (43.9%)	94 (46.6%)	53 (39.9%)	
Weight (Kg) (mean± SD)	47.5 ± 25.9	38.5 ± 17.1	61.9 ± 31.5	<0.001
Range	(13.4–167.7)	(13.4–80)	(21–167.7)	
Height (cm) (mean± SD)	144.2 ± 23.5	142.5 ± 23.9	147.3 ± 22.97	0.069
Range	(95–186.6)	(95–186.6)	(96–196.4)	
Body Mass Index (BMI) mean				
± SD	21.4 ± 6.9	18.0 ± 3.9	26.5 ± 7.6	<0.001
Range	(12.9–58.3)	(12.9–24.6)	(17.3–60.86)	
BMI percentile mean ± SD	65.7 ± 30.5	46.6 ± 24.9	94.7 ± 4.3	<0.001
Range	(1–99)	(1–84)	(85–99)	
Body Surface Area (BSA)(m2)				
mean ± SD	1.35 ± 0.46	1.22 ± 0.37	1.55 ± 0.5	<0.001
Range	(0.59–2.56)	(0.59–2.0)	(0.7–2.56)	

Overweight: BMI percentile equal or more than 85^th^ percentile.

* Includes Hispanics, Asian and those who refused to reveal their race.

P values are based on the comparison of normal weight group and overweight/obese group.

In 319 (95%) studies, the data used for analysis was a mean of three measurements for each study and in 16 (4.8%) it was a mean of two studies. The coefficient of variation (CV) for three measurements was 0.75% ± 1.0% (Mean ± SD) range 0–3.7%. For two measurements (two each in three combinations), the CV% was 0.58% ± 1.63%, 0.59% ± 0.93%, and 0.79% ± 1.9%, respectively. Only three studies had CV% of more than 5% and they were excluded from data analysis.

**Fig 2:**
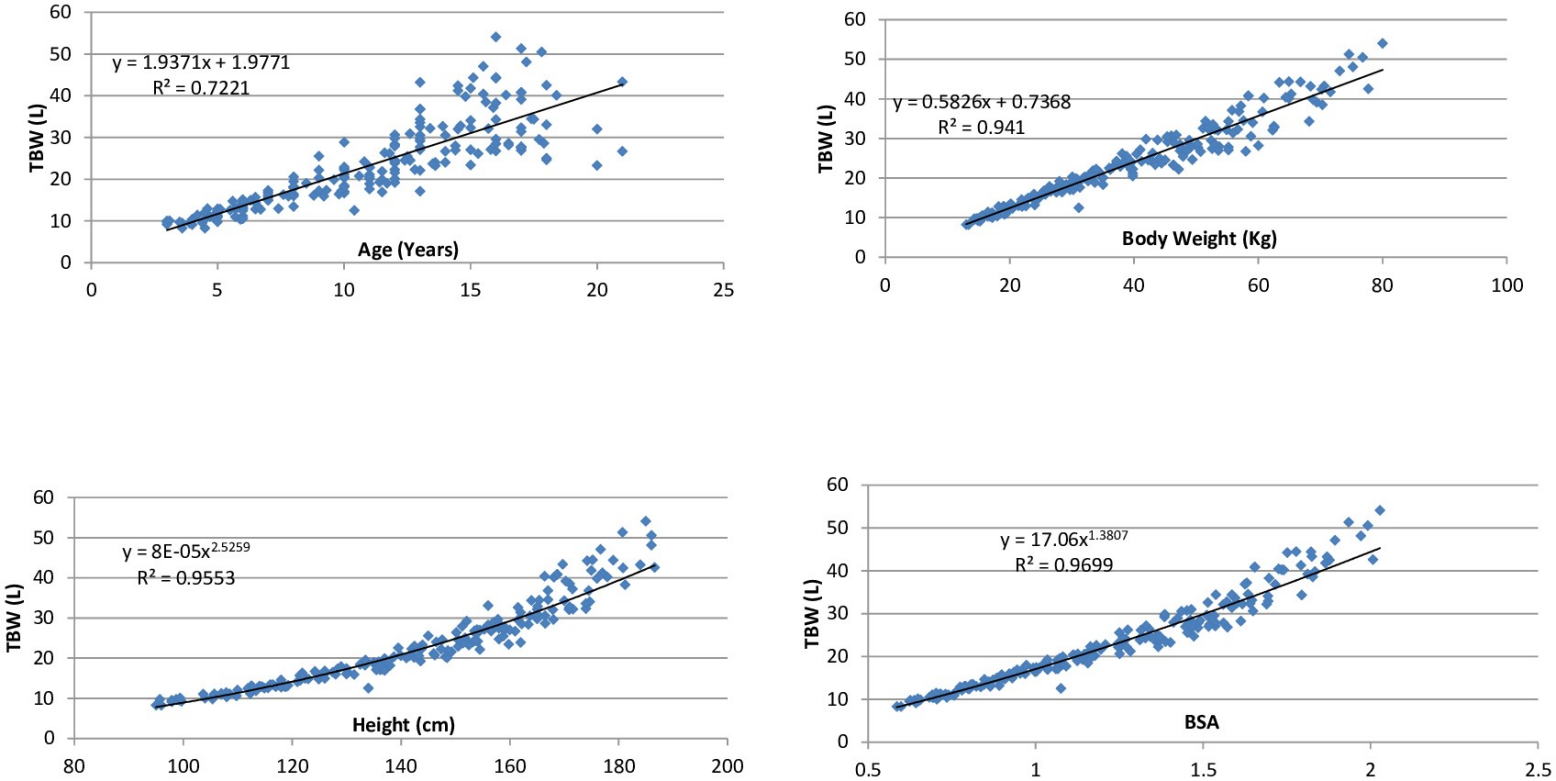
Correlation between total body water and age, weight, height, and BSA in normal weight individuals. TBW: Total Body Water; BSA: Body Surface Area in m^2^.

TBW according to the various age groups is shown in Table 2. Scatterplots of TBW individuals with normal weight (n = 202) in relation to their age, body weight, height, and body surface area (BSA) are shown in Fig 2. Separate simple linear regressions and R-squared values were obtained for age (72.2%), body weight (94.1%), height (95.5%), and BSA (97.0%).

**Table 2:** Total body water according to various age groups.

Age Groups	Male			Female		
	Normal Weight	Excessive Weight	P Value	Normal Weight	Excessive Weight	P Value
3–7 Years	12.5±2.5 (n = 23)	15.0±3.7 (n = 21)	0.011	12.2±2.2 (n = 36)	15.1±3.7 (n = 13)	<0.01
8–12 Years	20.7±4.6 (n = 32)	26.5±7.6 (n = 27)	<0.01	21.6±3.8 (n = 35)	25.3±5.8 (n = 25)	<0.01
13–17 Years	37.8±7.5 (n = 37)	45.1±8.7 (n = 21)	<0.01	28.4±4.3 (n = 30)	37.5±9.0 (n = 15)	<0.01
18–21 Years	42.0±1.7 (n = 3)	46.8±5.8 (n = 7)	0.21	27.5±4.1 (n = 6)	40.1±9.0 (n = 4)	0.015

Excessive weight: Overweight and obese.

**Fig 3:**
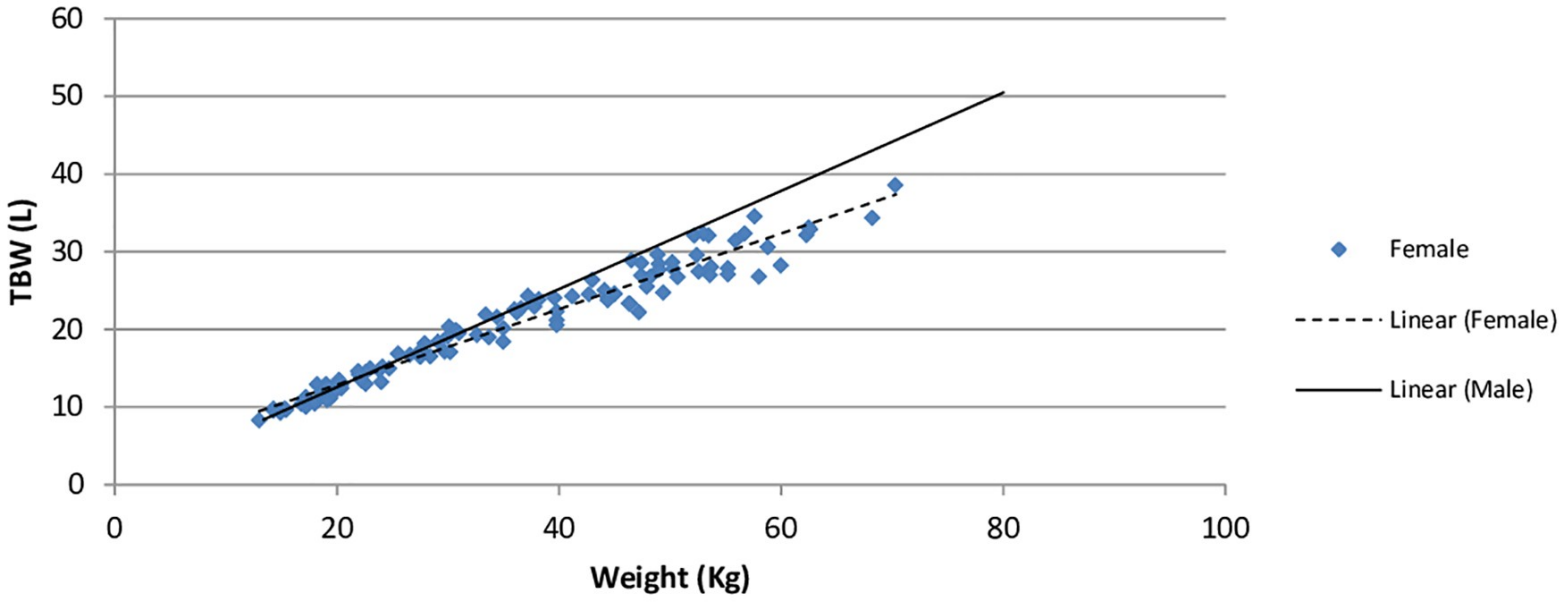
Total body water (TBW) in males and females with normal weight.

A scatterplot of TBW between genders of normal weight is shown in Fig 3. Females had a slightly higher R-squared value (94.4%); TBW = 6.878 + 0.359 (x) compared to males (92.9%); TBW = 5.056 + 0.456 (x). In terms of feasibility, both equations were highly predictive for both females and males with normal weight using BIA estimation. When controlling for body weight, age, BSA, and height males (97.3%) and females (97.8%) had almost identical R-squared values (97.3%). Results from the General Linear Model (GLM) revealed a significant (P<0.01) difference in the mean TBW in males (24.04, SE 0.20) versus females (22.47, SE 0.19) with normal weight. Covariates appearing in the model were evaluated at the following values: body weight = 38.57, height = 142.49, BSA = 1.22, age = 10.96. No significant mean differences in TBW were found between ethnicity groups.

**Fig 4:**
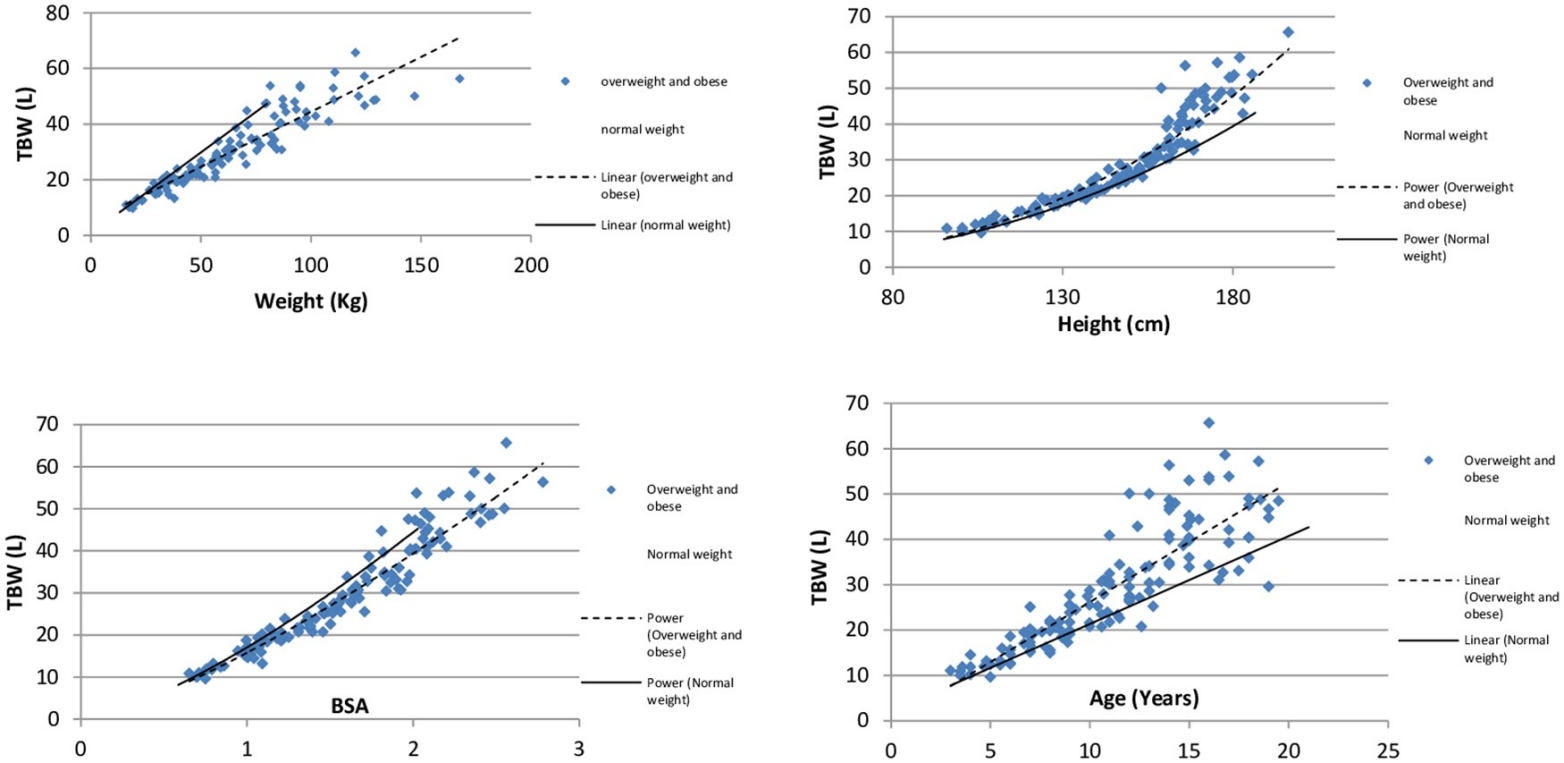
Total body water in overweight and obese individuals as compared to those with normal weight. TBW: Total Body Water; Overweight includes obese participants; BSA: Body Surface Area in m^2^.

Scatterplots of TBW in normal (n = 202) and individuals with excessive weight (n = 133) in relation to their age, body weight, body surface area, and height (BSA) are shown in Fig 4. Individuals with excessive weight had higher mean TBW (27.87, SE 0.368) for height and age as compared to individuals with normal weight (23.95, SE 0.298), P<0.001 (covariates age = 11.0, height = 144.2). However, the mean TBW for weight and BSA was lower in individuals with excessive weight (24.93, SE 0.37) as compared to individuals with normal weight (26.94, SE 0.287), P<0.001 (covariates weight = 47.5, BSA = 1.353).

The correlation coefficients of TBW with participant age, weight, height and BSA were (rounded) 0.72, 0.94, 0.96, and 0.97, respectively. The mean TBW (L) in males and females with normal as well as excessive weight was 26.52 L (SE) 0.21 and 24.46 L (SE) 0.22 (P<0.001), respectively; covariates appearing in the model were evaluated at the following values: Body Weight = 47.51, Height = 144.15, BSA = 1.35.

A comparison of our demographic data in children with normal weight with the two studies by isotope dilution methods that either published individual patient data (Cheek et al.) [14] or made it available to us on request (Dasgupta et al.) [15] is shown in Table 3. It reveals that there were no significant differences in all standardized median residual values predicting TBW between our data and that produced the two isotope dilution studies (BSA: p = 1.00; body weight: p = 0.93; height: p = 0.57; age: p = 0.87).

**Table 3:** Standardized residual median regression values for total body water by each single predictor by study investigator.

	Our Study	Dasgupta et al. [15]	Cheek et al. [14]	P-Value
BSA	0.08	0.03	0.04	1.00
Weight	0.11	-0.02	-0.05	0.93
Height	-0.15	0.03	0.11	0.57
Age	-0.05	-0.02	-0.13	0.83

BSA: Body surface area.

**Fig 5:**
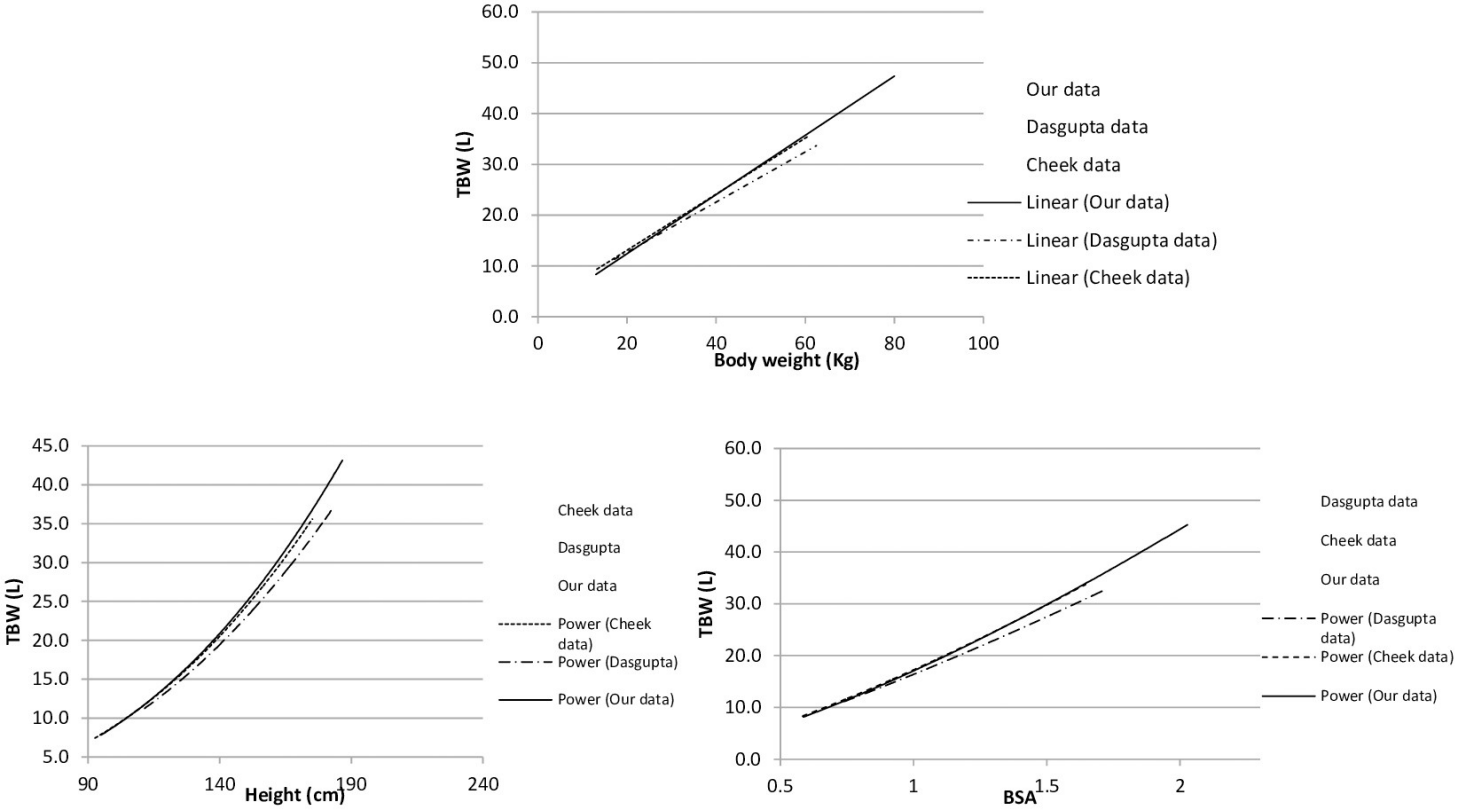
Comparison of regression line fits between bioimpedance and dilution methods. BSA: Body surface area Cheek et al^14^, Dasgupta et al^15^.

Fig 5 reveals that our data very closely replicated the scatterplot graphs by dilution methods of TBW. Data from all three investigations revealed that the relationship between TBW and body weight best fit a linear function but the relationship between TBW with height and TBW with BSA best fit a power curve function.

**Fig 6:**
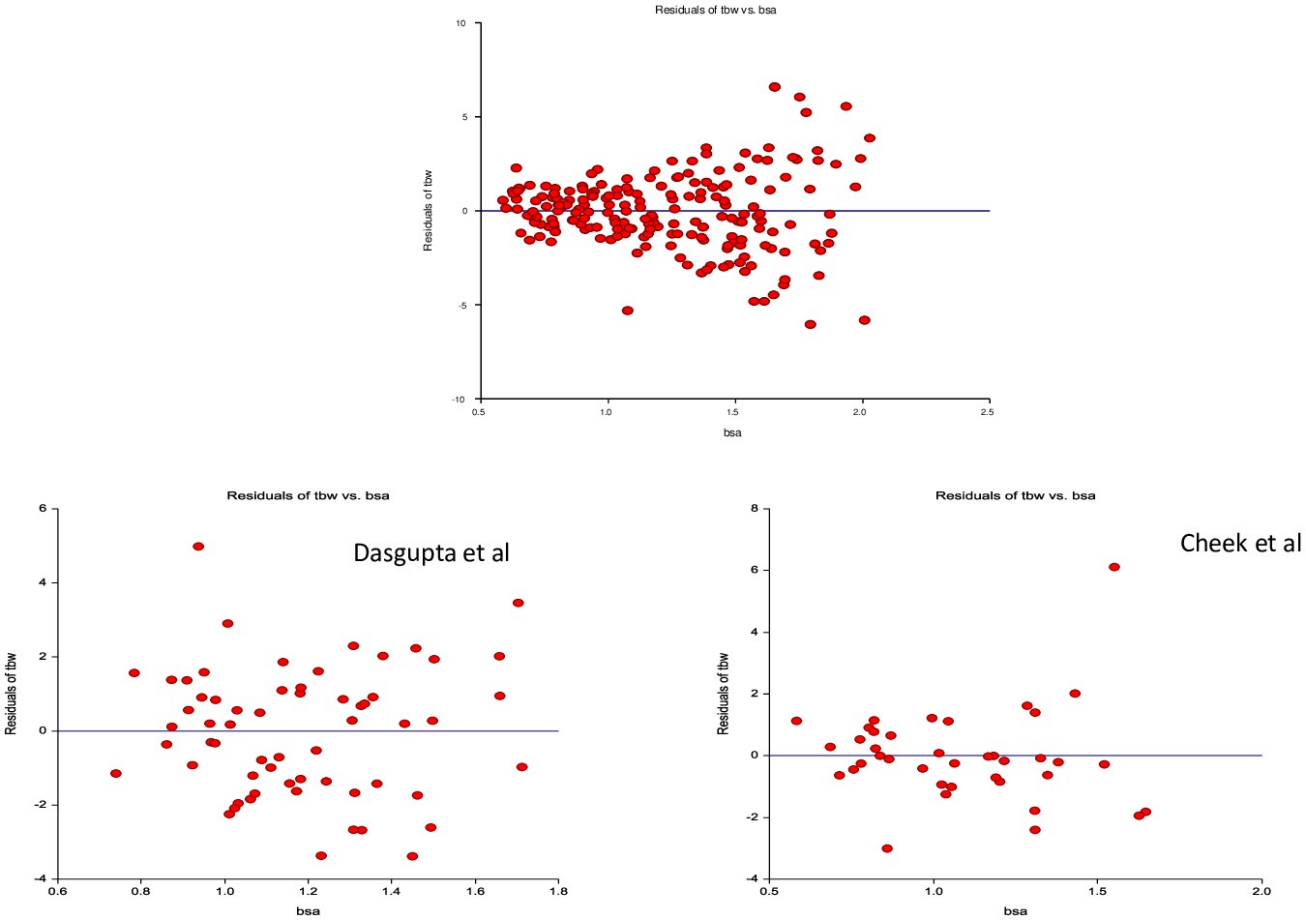
Comparison of regression residuals between bioimpedance and dilution methods. BIA: Bioimpedance analysis; TBW: Total body water; BSA: Body surface area; Studies by dilution method (Cheek^14^, Dasgupta^15^).

Fig 6 displays the regression residual plot graphs for TBW by BSA between our data and that produced by the two dilution methods. Residuals from the BIA method and the dilution method are not systematically different in range until approximately 15 years of age when a greater spread is seen in all predictive variables examined in the BIA graphs.

Accuracy of prediction statistics from the regressions from the three studies are shown in Table 4. R-squared values for predicting TBW with BSA, body weight, height, and age was 96% for the BIA method and 91% and 95% for the dilution method. The square root of MSE was slightly higher for the BIA method (2.05) than for the two dilution methods (1.79 and 1.58). Mean absolute percent error (MAPE) calculated from the BIA data (6.47) was between the two calculated using the dilution method (7.10 and 5.15).

**Table 4:** Accuracy of prediction statistics by study investigator.

	Our Study	Dasgupta et al. [15]	Cheek et al. [14]
R^2^	0.9610	0.9097	0.9535
Adjusted R^2^	0.9602	0.9032	0.9481
Mean Squared Error	4.1882	3.1892	2.4950
Square Root of MSE	2.0465	1.7858	1.5796
Mean Abs Pct Error*	6.473	7.104	5.151

*Mean Abs Pct Error: Average Absolute Percent Error.

## Discussion

BIA is the most practical method for TBW estimation in children in the outpatient clinic setting. Previously considered as less accurate, the introduction of a multi-frequency BIA has helped enhance its accuracy [16]. The major advantages of BIA over other methods of body water estimation are that the equipment is non-invasive, inexpensive, portable, easy to use, and takes five minutes to complete. Furthermore, the results are immediately available and it is possible to have multiple readings. In 1994, the Technology Assessment Conference Statement by the National Institutes of Health concluded that the BIA provides a reliable estimate of TBW in most conditions and in view of its ease of measurement, expense, safety, portability, and reproducibility is preferred over logistically complex techniques [17]. According to the European Society for Clinical Nutrition and Metabolism Guidelines, the BIA works well in healthy subjects and in patients with stable water and electrolyte balance [18–22].

A large number of studies have been done to validate BIA against reference techniques for the measurement of TBW and ECW [5, 8–10]. These studies have revealed a good overall agreement between dilution techniques and BIA in healthy children as well as adults [23–25], hospitalized elderly patients [26] pregnant women [27], diabetic patients [28], children with obesity [29], and during rehydration for cholera [30]. A study in healthy as well as malnourished children revealed that the BIA method was accurate within 4% of the mean body water measured by isotope dilution [31]. BIA’s accuracy in detecting changes in blood volume was also demonstrated in adults [32–34] and children on dialysis [16, 35–38].

Apart from the ease of data collection in children by BIA, our study revealed excellent reproducibility of BIA readings with a very low coefficent of variation between the three readings. A high reproducibility with <1% error on repeated measurements for TBW and ECW has been reported previously also [23, 39]. Our study revealed a significant correlation of body water content with height, weight and BSA, but not patient age. It showed a linear relationship with age and weight and a curvilinear relationship with height and BSA. This is similar to the study by Cheek et al. [14] that showed a linear relationship of TBW with weight and a curvilinear relationship with height, the latter because of growth spurt.

Males with normal weight in our study showed 9% more mean TBW as compared to females with normal weight. When combined with those with overweight and obesity, the males had 7.7% more TBW than females. The reported gender difference for TBW by dilution method in children is variable. It ranges from about 6% higher TBW in males aged 7–9 years [40] to about 15% in males aged 5–8 years [41]. The TBW increases with growth and our study revealed that the TBW was very similar in boys and girls with normal weight in 3–7 year age group and it was significantly lower (p<0.01) in females as compared to males in 18–21 year age group. For those with excessive weight, the TBW was similar in 3–7 year age group, but it did not show any significant difference (p 0.16) in 18–21 age group. However, the number of patients in 8–21 year age group in our study is very small and will need further validation.

Studies in adults have revealed that individuals with overweight and obesity have lower TBW for weight and hence are hypohydrated as compared to those with normal weight [42–44]. Very little has been published about weight related changes in TBW in children and the observations made are very similar to adults [29, 45, 46]. A weight related decrease in TBW in obesity is a result of relatively higher percentage of body fat in such individuals with a net decrease in fat-free mass and TBW for weight. Body fat has only 20–30 percent water as compared to about 70% in fat-free body mass [47]. In our study, individuals with excessive weight had 16.5% higher mean TBW for height and age and 7.4% lower TBW for weight and BSA as compared to normal weight individuals.

Body water measurement by BIA is affected by multiple factors. These include room temperature, electrode placement, skin temperature, posture, recent physical activity, full bladder, changes in plasma osmolality or sodium concentration, hydration status, consumption of food and beverages, conductance of examination table, ethnicity, menstruation, and underlying medical conditions [5, 17, 48–51]. The accuracy of measurement is also affected by the variability of prediction equations for a particular patient population [26]. In our study, we overcame some of these limitations by using strict study inclusion/exclusion criteria and standardizing methods for data collection as elaborated previously, and by using the same BIA machine for all participants. Our study cohort consisted of healthy children and young adults with no suspected systemic or fluid and electrolyte abnormality.

Our study explored the feasibility of BIA data collection in routine pediatric outpatient clinic setting. Instead of validating our data by comparing it with a reference method, which would have been impossible in our setting, we compared our data with the two studies that either published individual patient data [14] or made it available to us on request [15]. Our results showed no significant mean differences in TBW with the former but a significant difference with the latter. It is interesting to note that the two studies, both by dilution method, showed significant difference amongst themselves, which might be indicative of some inherent limitations even with indirect method for body water estimation. The study cohort for these studies, including ours, were not age, weight, race or gender-matched, which may explain the differences. However most of the individuals, 90% in one study [15] and 93% in the other study [14], had normal weight and hence comparable to our study cohort with normal weight.

As in adults, hypohydration in children and young adults with excessive weight has clinical implications. These include increased risk of dehydration, assessment and management of dehydration, calculation of volume of distribution for medications, and management of renal failure, including dialysis. In 2004, The European Society of Clinical Nutrition and Metabolism (ESPEN) recommended to use BMI for TBW measurement only for BMI between16-34. Higher BMI in some of our patients could be a study limitation. However, studies published after the publication of ESPEN guidelines have reported that BIA accurately estimates TBW in overweight and obese subjects [52, 53] and this may be due to an increasing use of multi-frequency BIA for data collection. Not having a concurrent reference method to validate BIA results in our study cohort could be seen as a study limitation. However, our study results are consistent with observations made in children, who were studied by dilution methods. Our observations are based on a single-center study cohort and a larger multi-center study will be needed to see if the results are any different in a national cohort. Being an observational, exploratory and a pilot study, we did not do power analysis for the estimation of our sample size. However, a post hoc power analysis using the SE of TBW and BSA, which was 0.287, with a 95% level of confidence and the margin of error set at 3.1%, the sample size would be n = 333.

In conclusion, children and young adults with excessive weight are hypohydrated, which may increase their risk of dehydration. Further studies are needed to evaluate the clinical significance of hypohydration in this population.

## Supporting information

S1 Data (XLSX)
